# Little Old Lady, Me? Modern Cinematic Representations of Older Women and Challenging the Narrative of Decline

**DOI:** 10.1093/geroni/igaf122.3351

**Published:** 2025-12-31

**Authors:** Neasa Fitzpatrick

**Affiliations:** Trinity College Dublin, Waterville, Kerry, Ireland

## Abstract

Introduction Ageing discourse is dominated by a ‘narrative of decline’ that leaks into popular culture. Women are disproportionately affected by this and older women have been under-represented in cinema. However their visibility has increased in the past two decades. We explore the representations of older women in modern cinema and their relationship to the narrative of decline and other ageing stereotypes. Methods Films of the past two decades with female leads over the age of 65 were reviewed. Focus was directed on popular and/or acclaimed films in mainstream and independent cinema. Characterisations of older women were analysed for common themes and patterns. Typical characterisations were identified and analysed in the context of the ‘narrative of decline’. Results Two stereotypical portrayals of older women were identified and subsequently described: 1. ‘Romantic rejuvenation’ where the older woman reclaims youthful attributes through romantic affairs, and 2. ‘The passive problem’ in which the older woman has a degenerative disability a that poses challenges and burdens to her spouse. Both representations were found to reinforce the narrative of decline. A third representation challenged this narrative: ‘The “Old Woman” in her own words’ – authentic, engaging depictions of older women from older female filmmakers. Conclusion Rhetoric around ageing women remains entrenched in a narrative of decline, framed in modern cinema as something to avoid or lament. The agency of older women is underestimated, which can have implications for health and social care. When voices are given to older women, we can appreciate their rich inner lives.

